# A non-parametric analytic framework for within-host viral phylogenies and a test for HIV-1 founder multiplicity

**DOI:** 10.1093/ve/vez044

**Published:** 2019-11-04

**Authors:** Eric Lewitus, Morgane Rolland

**Affiliations:** 1 U.S. Military HIV Research Program (MHRP), WRAIR, 503 Robert Grant Avenue, Silver Spring, MD, USA; 2 Henry M. Jackson Foundation for the Advancement of Military Medicine, Inc., 6720A Rockledge Dr, Bethesda, MD, USA

**Keywords:** phylogenetics, HIV-1, methodology, Laplacian, vaccine, HIV-1 founder multiplicity

## Abstract

Phylogenetics is a powerful tool for understanding the diversification dynamics of viral pathogens. Here we present an extension of the spectral density profile of the modified graph Laplacian, which facilitates the characterization of within-host molecular evolution of viruses and the direct comparison of diversification dynamics between hosts. This approach is non-parametric and therefore fast and model-free. We used simulations of within-host evolutionary scenarios to evaluate the efficiency of our approach and to demonstrate the significance of interpreting a viral phylogeny by its spectral density profile in terms of diversification dynamics. The key features that are captured by the profile are positive selection on the viral gene (or genome), temporal changes in substitution rates, mutational fitness, and time between sampling. Using sequences from individuals infected with HIV-1, we showed the utility of this approach for characterizing within-host diversification dynamics, for comparing dynamics between hosts, and for charting disease progression in infected individuals sampled over multiple years. We furthermore propose a heuristic test for assessing founder heterogeneity, which allows us to classify infections with single and multiple HIV-1 founder viruses. This non-parametric approach can be a valuable complement to existing parametric approaches.

## 1. Introduction

Molecular rates of evolution impact patterns of virulence and viral transmission (Duffy, Shackelton, and Holmes 2008; Ho et al. 2011). High mutation rates, large population sizes, and small genomes typical of viruses lead to heterochronous rates of evolution (Peck and Lauring 2018), often characterized as diminishing returns on strong purifying selection (Sharp et al. 2001; Holmes 2003). Approaches to detecting these patterns of selection comprise pairwise diversity estimates (Sánchez-DelBarrio et al. 2003), model-based inferences of substitution rates (Pond and Frost 2005; Lemey et al. 2007), and illustrative measures, such as codon frequencies (Kumar, Tamura, and Nei 1994). Although these form much of the foundation of our understanding of viral evolution, they nonetheless present some limitations for inferring and comparing within-host diversification dynamics (Ratmann et al. 2017; Mitov and Stadler 2018). These limitations frequently stem from the general drawbacks of applying model-based approaches to complex data and from inherent difficulties in directly comparing incompatible models across individuals (Duchêne, Ho, and Holmes 2015).

We present the spectral density profile of the modified graph Laplacian (MGL) as a framework for characterizing and comparing virus evolution within and across hosts (Lewitus and Morlon 2016a). This approach allows the user to directly interpret the within-host diversification dynamics of a virus through interpretable evolutionary parameters and to compare those parameters between hosts. As such, it relates the molecular evolution of a virus at the nucleotide level to its diversification dynamics throughout a population and therefore provides a framework for realizing and interpreting diversification trends, clusters, and deviants across a set of sampled individuals or cohorts. Importantly, this approach is non-parametric and therefore is fast and does not rely on model assumptions. When compared with some previous methods (Robinson and Foulds 1981; Amenta and Klingner 2002; Hillis, Heath, and John 2005; Kendall and Colijn 2016), the MGL approach allows for direct comparisons of entire trees, even when there are different tip numbers and labels. We showed how the spectral density profile of the MGL may be interpreted in terms of viral diversification dynamics using simulated alignments and phylogenetic tree reconstruction. To demonstrate the utility of the approach for hypothesis-testing and unbiased data exploration, we analyzed HIV-1 sequences sampled from participants infected during the RV144 trial (Rerks-Ngarm et al. 2009), developed a heuristic test for classifying founder pool heterogeneity in HIV-1 *env* sequences obtained from acutely infected individuals (Keele et al. 2008), and charted the diversification dynamics associated with HIV-1 evolution over several years (Shankarappa et al. 1999) with time-stepped profiles.

## 2. Results

### 2.1 Formulating the MGL for a viral phylogeny

**Figure 1.**
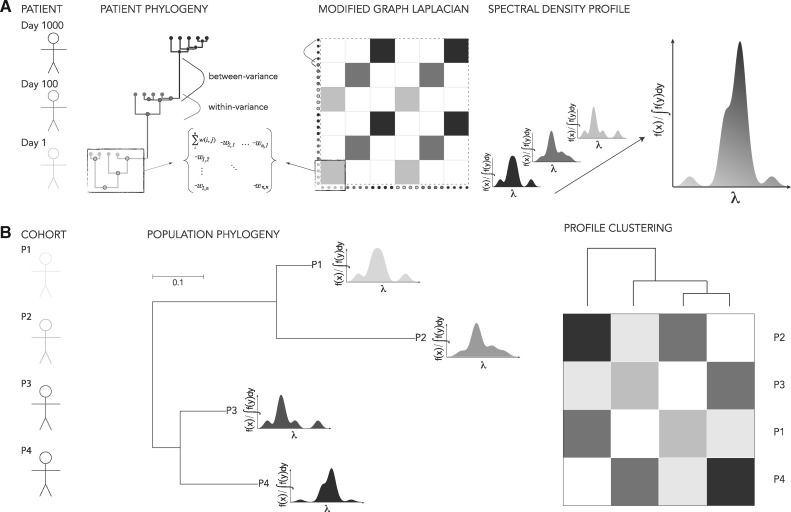
Schematic of the spectral density profile for (A) an individual-level phylogeny and (B) population-level phylogeny. In (A), a phylogeny is constructed from viral sequences sampled from a participant at three time-points; the MGL of the phylogeny captures the topology generated from genetic dissimilarity sampled from the same time-point (within-variance) and the genetic dissimilarity between time-points (between-variance); the eigenvalues, *λ*, computed from the MGL used to plot the spectral density profile represent the cumulative pattern of between- and within-variance of genetic dissimilarity of the individual phylogeny. In (B), spectral density profiles computed from multiple participants can be represented on a population-level phylogeny according to the genetic dissimilarity of consensus sequences for each participant. Spectral density profiles can be clustered across participants based on the amount of divergence between profiles.

The spectral density profile of the MGL allows for direct comparisons of patterns of phylogenetic diversification (Lewitus and Morlon 2016a,b). The Laplacian graph, Δ, is computed for the distance matrix of the reconstructed phylogeny of within-host sampled viral sequences,

(1) $$\Delta (i,j)=\{\begin{array}{cc} \sum w(i,j), & \text{if }i=j.\\ -w(i,j), & \text{otherwise}. \end{array}$$

where each off-diagonal cell is the negative of the distance between nodes *i*, *j* and each diagonal cell is the sum of distances in row *i*. The eigenvalues, *λ*, calculated from the graph define the connectivity of the phylogeny, such that larger *λ* indicate sparse connectivity and smaller *λ* indicate dense connectivity (Noh and Rieger 2004; Banerjee and Jost 2009). Here the definition of connectivity is contingent on the phylogeny—for example, an ultrametric tree will define connectivity in terms of time, whereas a non-ultrametric tree may define connectivity in terms of number of nucleotide substitutions (Fig. 1A). The spectral density profile is then constructed by convolving *λ* with a smoothing function,

(2) $$f(x)=\sum_{i=1}^{n}{\left(2\pi \sigma^{2}\right)}^{-1/2}e^{\left(\frac{-|x-\lambda_{i}{|}^{2}}{2\sigma^{2}}\right)}$$

so that the profile is plotted for f(x)/∫f(y)dy as a function of *λ*. Profiles for different sets of viral sequences (or different phylogenetic builds of the same sequences) can then be clustered based on their Jensen-Shannon distances (Endres and Schindelin 2003) and an optimal number of supported clusters determined by, for example, partitioning around medoids (Reynolds et al. 2006) (Fig. 1B, see Section 5). The spectral density profile can be sufficiently summarized using statistics that represent different aspects of the topology of the phylogeny: the principal eigenvalue ($\lambda^{*}$) is a measure of the longest path through the phylogeny and so estimates the upper-bound of evolutionary change present in the sample; skewness (*ψ*) reflects the proportion of long *versus* short branching-events, where long and short are relative to the distribution of branch-lengths in the phylogeny; and peak height (*η*) indicates the heterogeneity of branching-events, where lower *η* means more heterogeneity (Lewitus and Morlon 2016a). The eigengap, which is defined as the position of the largest discrepancy between two eigenvalues when the eigenvalues are ranked in descending order, is a unique feature of the Laplacian graph and is a signifier of the number of disconnected sets of branches (due, e.g., to a shift in diversification rate) in the phylogeny (Von Luxburg 2007; Shen and Cheng 2010; Lewitus and Morlon 2016a). Each statistic can be interpreted in terms of the diversification dynamics of the virus, as we demonstrate below; and therefore, individual and clusters of phylogenies can be characterized by their summary statistics, including a classification scheme for founder heterogeneity.

Code for computing the spectral density profile of the MGL of phylogenies can be found in *RPANDA* (Morlon et al. 2016) and *R* code for applying a test of founder heterogeneity is available at https://www.hivresearch.org/publication-supplements. Alignments from Keele et al. (2008), Rolland et al. (2012) and Shankarappa et al. (1999) can be found at https://www.hiv.lanl.gov/content/sequence/HIV/SI_alignments/datasets.html.

### 2.2 Interpreting the MGL at the molecular level

**Figure 2.**
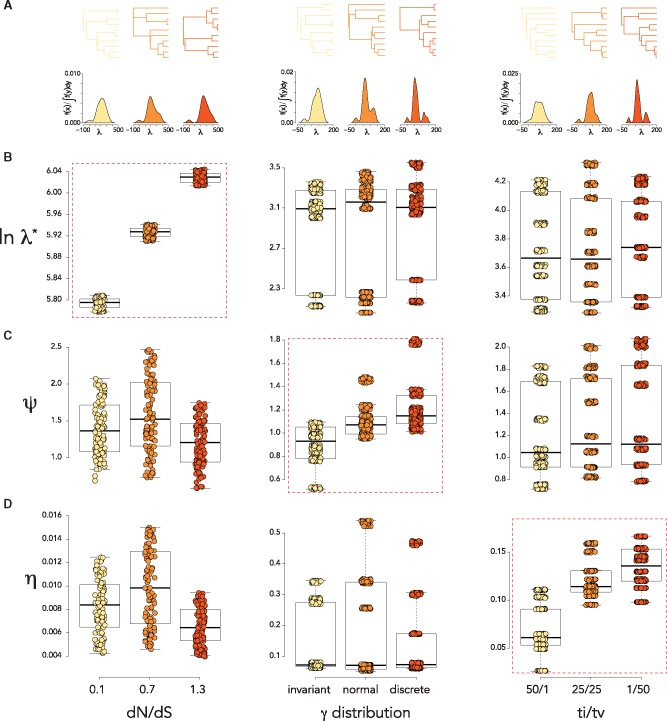
Interpreting the MGL at the molecular level. (A) Representative phylogenies and spectral density profiles of the parameters in (B–D). (B-D) boxplots for $\lambda^{*}$, *ψ*, and *η* for alignments simulated under various (left) dN/dS, (middle) *γ* distributions, and (right) ti/tv. Dashed red boxes indicate significant differences (*P* < 0.01) between all three parameter values.

The significance of the spectral density profile was validated by constructing phylogenies from sequences simulated under various scenarios of molecular evolution. We predicted that each summary statistic would be sensitive to a particular generative mechanism, as each of these generative mechanisms would have a particular effect on the phylogeny. We found that trees simulated under different non-synonymous/synonymous substitution rates (dN/dS) could be distinguished by their $\lambda^{*}$ (Fig. 2A). Higher levels of variance in the distribution of rates, ranging from different rates at a few discrete sites (strong rate heterogeneity) to similar rates across all sites (weak rate heterogeneity) (Nielsen and Yang 1998), produced trees with higher *ψ* values (Fig. 2B). In addition, we observed that higher transition/transversion (ti/tv) rates, which typify fewer substitutions detrimental to fitness and signifies mutational fitness in HIV-1 (Lyons and Lauring 2017), produced trees with lower *η* values (Fig. 2C). We also compared maximum pairwise genetic dissimilarity between simulated sequences in each scenario; this was less effective than $\lambda^{*}$ and *ψ*, respectively, in distinguishing between samples simulated under different dN/dS and rate heterogeneity and ineffective in distinguishing differences in ti/tv rates (Supplementary Fig. S1).

**Figure 3.**
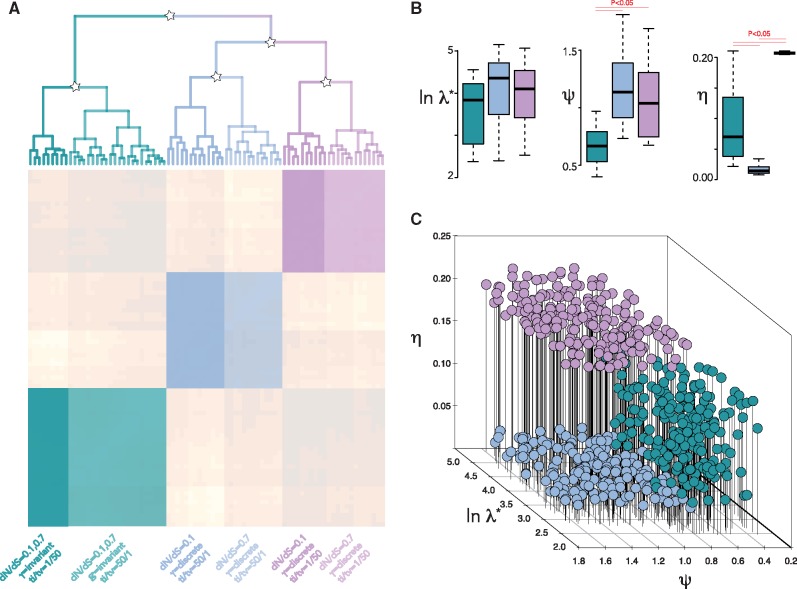
Unbiased clustering of spectral density profiles. (A) Heatmap and hierarchical clustering of spectral density profiles for trees simulated under eight evolutionary scenarios. Stars indicate bootstrap support ≥0.9. The color scheme (cyan, blue, and violet) distinguish the three clusters determined by partitioning around medoids. Shades of the same color further distinguish clusters with divisions identified by hierarchical clustering using bootstrap probability support. Hierarchical clustering is not shown below a threshold value of 2. (B) Boxplot of spectral density profile summary statistics for trees belonging to each cluster based on medoid partitioning. (C) Simulated trees within each cluster plotted into multidimensional space defined by spectral density profile summary statistics.

We compared spectral density profiles for trees simulated under permutations of all three parameters: dN/dS = 0.1, 0.7; *γ* distribution = discrete, invariant; and ti/tv = 1/50, 50/1. Using Jensen-Shannon distances between profiles, we identified three clusters with average silhouette widths > 0.7 (where widths > 0.5 indicate robust cluster assignment; Rousseeuw 1987) by partitioning around medoids, each of which could be further broken up into two clusters with bootstrap probability > 0.9 (Fig. 3A). By comparing spectral density profile summary statistics (Fig. 3B) and plotting the three clusters into a multidimensional space (Fig. 3C), we found that the trees were primarily distinguished by a combination of differences in *ψ* and *η*, reflecting the simulated differences in the *γ* distribution and ti/tv rates, whereas differences in dN/dS proved a less influential distinction.

We found the above processes could also be distinguished with time-scaled trees constructed using a Bayesian approach (Drummond and Rambaut 2007). The scale of inferred spectral density profiles was positively shifted with respect to the maximum-likelihood trees, underscoring the importance of comparing trees of similar build.

### 2.3 Interpreting the MGL for longitudinal samples

Within-host viral phylogenies are often sampled at multiple times post-infection. High mutation rates and frequent selective sweeps result in phylogenies that present with distinctly ladderized topologies (Fig. 1A; Shankarappa et al. 1999; Rambaut et al. 2004). We tested the effect of (1) elapsed time and (2) changes in diversification rate between sampling on the spectral density profile using simulated time-scaled trees.

**Figure 4.**
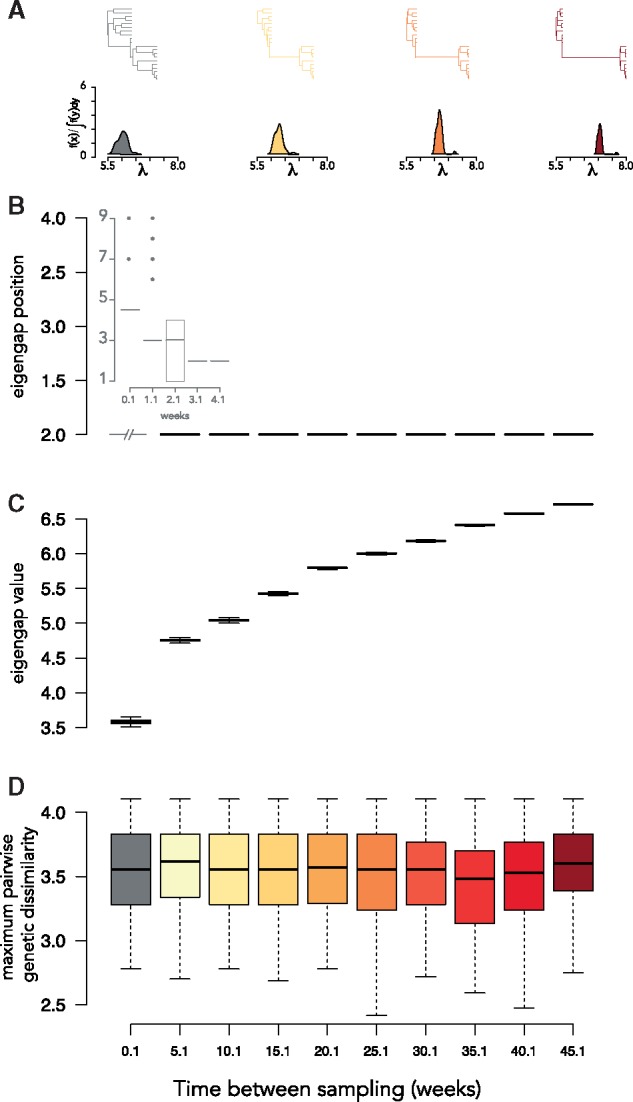
Interpreting the MGL for longitudinal samples: elapsed time. (A) Representative phylogenies and spectral density profiles for sequence data sampled at two time-points for increasing elapsed time between samples. Boxplot of the (B) eigengap position (i.e. inferred number of clusters), (B, inset) eigengap position for weeks 0.1–4.1, (C) the eigengap value (i.e. $\lambda_{i}-\lambda_{i+1}$), and (D) the maximum pairwise genetic dissimilarity over simulations of sequence data sampled at two time-points with increasing elapsed time between samples.

**Figure 5.**
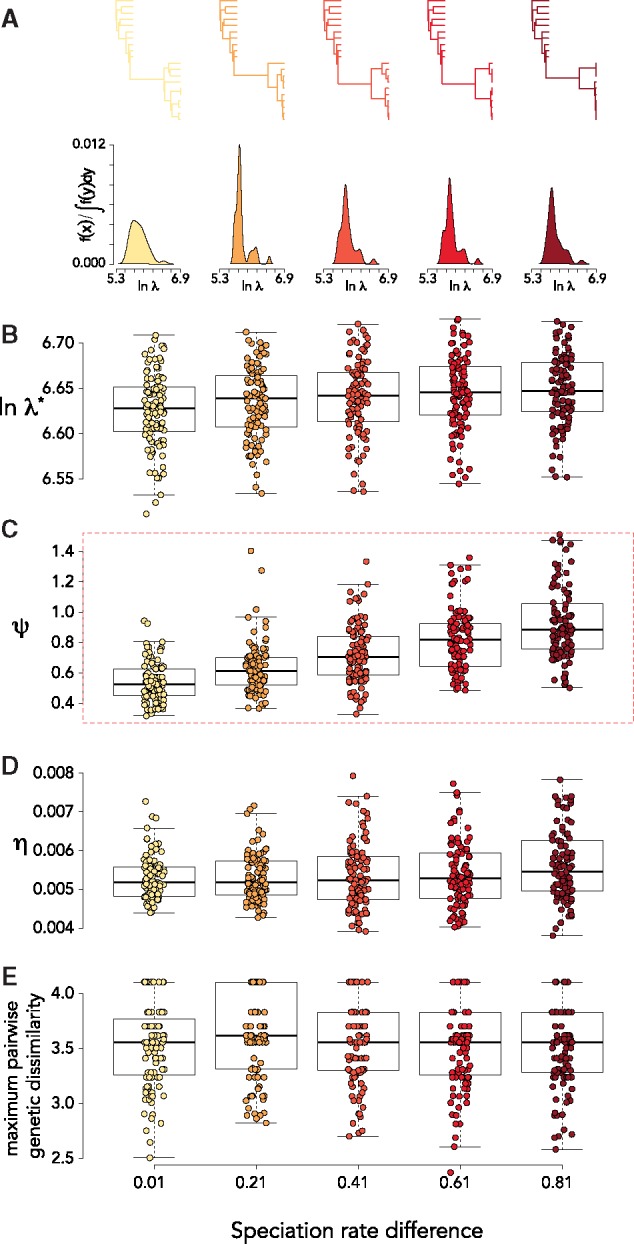
Interpreting the MGL for longitudinal samples: speciation rate differences. (A) Representative phylogenies and spectral density profiles for sequence data sampled at two time-points with increasing speciation rate differences between samples. Boxplot of (B) $\lambda^{*}$, (C) *ψ*, (D) *η*, and (E) maximum pairwise genetic dissimilarity over simulations of sequence data sampled at two time-points with increasing differences in speciation rate between samples. The dashed red box indicates significant pairwise differences in mean values (*P* < 0.01) between all groups.

(1) We found that the eigengap consistently identified two clusters when the simulated time between sampling was ≥3 weeks (Fig. 4A). The value of the eigengap was positively correlated with the time between sampling (Fig. 4B). Maximum pairwise genetic dissimilarity was unaffected by changes in the time between sampling (Fig. 4C). (2) When we held the time between sampling constant (10 weeks) and increased the difference in diversification rate between the first and second sampling, we found this was positively correlated with *ψ* ($y∼0.5x,R^{2}=0.99,P<6e-5$) (Fig. 5). There were minor positive effects on $\lambda^{*}$ ($y∼0.003x,R^{2}=0.89,P<0.01$), and *η* ($y∼0.0002x,R^{2}=0.91,P<0.01$) and no effect on maximum pairwise genetic dissimilarity (*P* = 0.87; Fig. 5).

#### 2.3.1 Example 1: Hypothesis-testing and exploration of within-host phylogenetic diversification in the RV144 cohort

**Figure 6.**
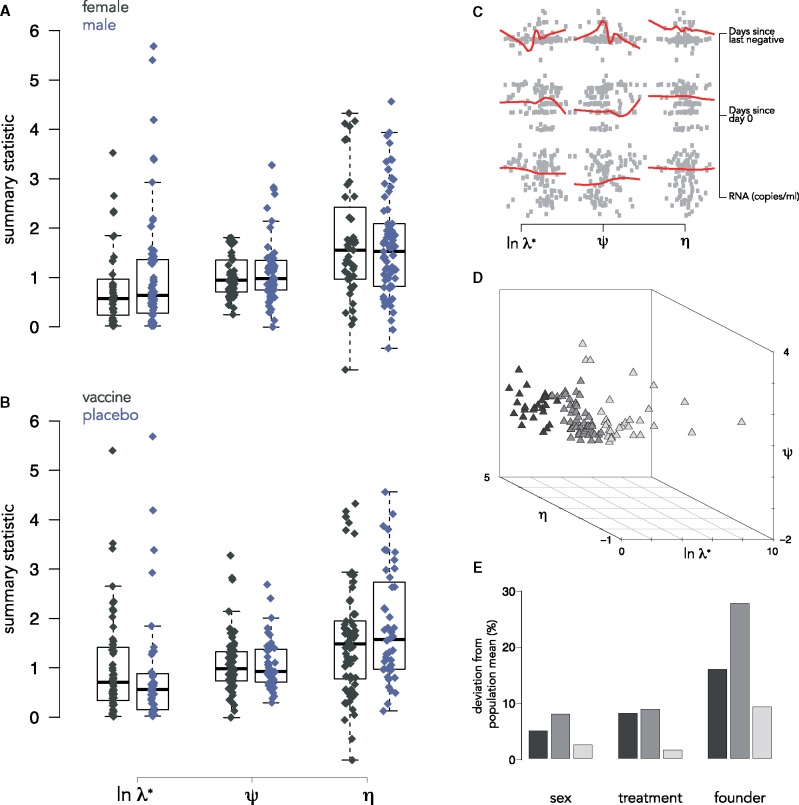
Hypothesis-testing on RV144 participants. Boxplot of spectral density profile summary statistics for (A) female and male infected individuals and (B) infected individuals administered a vaccine or a placebo in the RV144 trial. Outliers removed. (C) Pairwise plots and loess fits for different infection factors as functions of spectral density profile summary statistics for all infected individuals in the RV144 trial. (D) Multidimensional plot of phylogenies for all subtype CRF01_AE infected individuals from the RV144 trial. Points are shaded according to unbiased partitioning around medoids. (E) Barplot showing the % deviation of composition of infection factors of each cluster from the population mean (shades correspond to (D)).

To illustrate the utility of comparing and characterizing within-host viral diversification, we analyzed HIV-1 sequences sampled at diagnosis from individuals infected with HIV-1 CRF01 AE during the RV144 vaccine efficacy trial. We previously showed that HIV-1 genomes did not differ between individuals who were administered the vaccine or a placebo in terms of sequence diversity, divergence from the vaccine, or regarding the proportion of infections with multiple founders; however, there were significant amino acid differences between the groups at sites known to be targeted by antibodies elicited by the RV144 vaccine (Rolland et al. 2012). We constructed phylogenies for each sample based on nucleotide divergence and computed spectral density profiles from their MGLs. Using the metadata associated with the cohort, we tested for effects of sex, treatment, as well as the relationship between spectral density profile summary statistics and different infection factors. Using one-sample t-tests, we found no significant effect of sex (T<1,P>0.1; Fig. 6A) or treatment (T<1,P>0.1; Fig. 6B); nor of any of the infection factors on spectral density profile summary statistics (R<0.01,P>0.1; Fig. 6C).

We clustered the phylogenies based on Jensen-Shannon distances between their spectral density profiles. We identified three clusters (Fig. 6D) with an average silhouette width of 0.52. The composition of the clusters in terms of the infection factors associated with individuals in each differed slightly from the population mean for sex and treatment, but was most dramatically different for founder heterogeneity: in Cluster 1, participants with a heterogeneous founder pool constituted 16% of phylogenies; in Cluster 2, it constituted 23%; and in Cluster 3, it constituted 75% (Fig. 6E).

#### 2.3.2 Example 2: A heuristic test for founder pool heterogeneity

**Figure 7.**
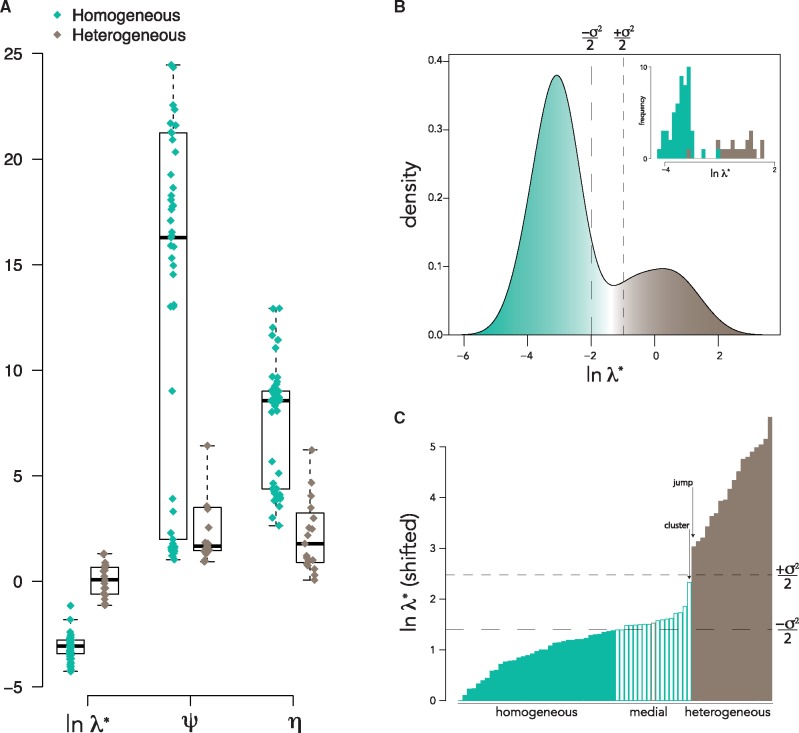
A heuristic test of founder heterogeneity.(A) Boxplot of spectral density profile summary statistics for acutely infected participants with founder homogeneity (green) and heterogeneity (brown) (Keele et al. 2008). (B) Density plot of $\text{ln}\lambda^{*}$ for all participants, nominally colored to show which tails of the distribution are predominantly occupied by participants with founder homogeneity and heterogeneity (see Supplementary Table S1). (B, inset) Histogram of $\text{ln}\lambda^{*}$ for individuals with founder homogeneity and heterogeneity. (C) Barplot of ranked $\text{ln}\lambda^{*}$, adjusted so that the minimum value is zero. Filled colors represent the inferred classification based on the principal eigenvalue test of founder heterogeneity using the median method (and inferred thresholds for the jump and partition methods are indicated with arrows); border colors represent the classification given in (Keele et al. 2008). $\pm \sigma^{2}/2$ of the median are shown with dashed lines on (B and C).

We used sequence data from Keele et al. (2008) to test the effect of founder pool heterogeneity on spectral density profile summary statistics. We constructed maximum-likelihood trees with *env* alignments from fifty-three participants with low-diversity sequences that conform to infections established by a single HIV-1 founder variant (i.e. homogeneous founder populations) and nineteen participants with more diverse sequences that correspond to infections established by multiple, related founder variants (i.e. heterogeneous founder populations; Keele et al. 2008). We found distinguishable patterns of diversification for the two groups. There were significant differences in mean values for $\lambda^{*}$ (T=12.69,P=1.12e−11; Fig. 7A). Significantly different distributions for homogeneous and heterogeneous groups for $\lambda^{*}$ (D=0.95,P=6.44e−15) resulted in disproportionate representation of participants with homogeneous founders in the left tail and heterogeneous founders in the right tail of the distribution of all participants (Fig. 7B). Specifically, 70 and 96% of homogeneous founders were to the left of the median and $+\sigma^{2}/2$ of the median of the distribution, respectively; and 100% of heterogeneous founders were to the right of the median of the distribution. The ‘jump‘ and ‘partition’ methods identified thresholds within $+\sigma^{2}/2$ of the median (Fig. 7C). We found that $\lambda^{*}$ was likewise effective when analyzing time-scaled trees, wherein 67 and 89% of homogeneous founders were to the left of the median and $+\sigma^{2}/2$ of the median of the distribution, respectively; and 92 and 100% of heterogeneous founders were to the right of the median and $-\sigma^{2}/2$ of the median of the distribution, respectively (Supplementary Fig. S2). We therefore can define a heuristic test for founder heterogeneity, wherein phylogenies with $\text{ln}\lambda^{*}<\text{ln}\lambda_{\text{median}+n\sigma^{2}}^{*}$ are classified as homogeneous/single founders and phylogenies with $\text{ln}\lambda^{*}>\text{ln}\lambda_{\text{median}+n\sigma^{2}}^{*}$ are classified as heterogeneous/multiple founders. Alternatively, homogeneous and heterogeneous founders can be distinguished using the ‘jump‘ or ‘partition‘ method, which identify thresholds consistent with the median technique. Perhaps more valuably, the distribution of $\lambda^{*}$ can be used to define the spectrum of founder heterogeneity within a sample, where medial assignation can be given to participants that fall between $\pm n\sigma^{2}$ of the median (Fig. 7C and Supplementary Table S1). Notably, there were no significant differences in mean phylogeny size (here defined by the number of tips) between homogeneous and heterogeneous groups (T=1.68,P>0.10).

#### 2.3.3 Example 3: Charting phylogenetic diversification of HIV-1 disease progression

**Figure 8.**
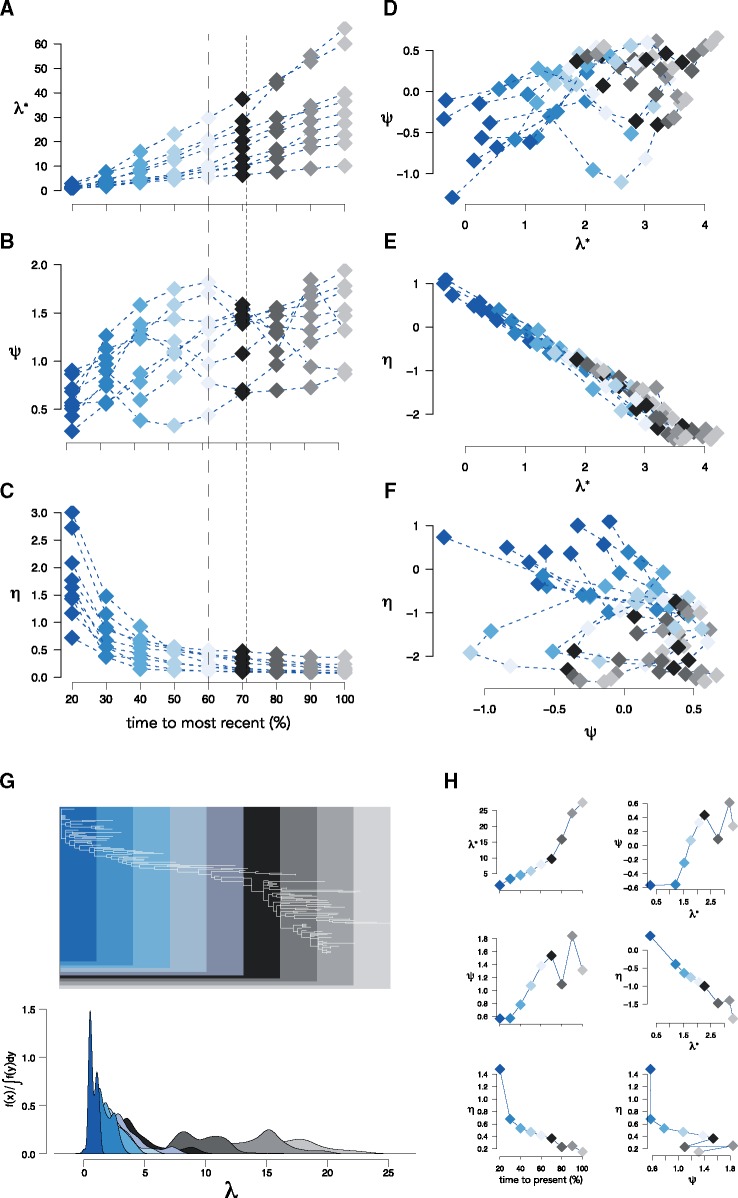
Tracking disease progression with the spectral density profile. Spectral density profile summary statistics for time-slices of HIV-1 phylogenies from nine individuals. (A–C) Summary statistics computed at each time-slice and (D–F) pairwise plots of summary statistics for each individual. Colors are coordinated with time-slices: 20% of time from root to present (dark blue); 100% time from root to present (lightest grey). Estimated diversity and divergence peaks from (Shankarappa et al. 1999) are shown as dashed and dotted lines, respectively, in (A–C). (G) The reconstructed HIV-1 phylogeny for Participant 1 showing the portion of the tree encapsulated by each time-slice. (H) Summary statistics through time and pairwise plots of summary statistics for Participant 1.

Phylogenetic diversification is a cumulative process: the diversification of an infection at time *t* = *t_n_* is measured as the sum of diversification events at $t\leq t_{n}$. We may, therefore, understand something about the progression of disease if we look at how diversification of an infection accumulates over time. We explored this using sequences from nine HIV-1-infected males who were sampled over 6–12 years as part of the Multicenter AIDS Cohort Study (Kaslow et al. 1987; Shankarappa et al. 1999). We constructed phylogenies for each individual. We then sliced each phylogeny at equally spaced time-points from the stem and computed the spectral density profile for each slice. For all individuals, $\lambda^{*}$ increased with each slice (Fig. 8A);*ψ* increased, too, although it oscillated from slice to slice (Fig. 8B); and *η* decreased exponentially, with an elbow roughly halfway between the stem and the present (Fig. 8C). Notably, the rate at which each summary statistic, particularly $\lambda^{*}$, changed through time was different for each individual, which is indicative of idiosyncratic disease progression between individuals. Likewise, the relationships between summary statistics at each slice was unique to each individual: *ψ* tended to increase as a function of $\lambda^{*}$, although not monotonically for each individual (Fig. 8D): *η* decreased as a monotonic function of $\lambda^{*}$ for all individuals (Fig. 8E), suggestive of a governing dynamic; and the relationship between *η* and *ψ* was inconsistent across individuals and, with one exception (Participant 9: $R^{2}=0.98,P<0.01$), uncorrelated (Fig. 8F).

Given these differences in the accumulation of diversification patterns between individuals, the spectral density profile of within-host phylogenies reconstructed for different time-slices can be used to understand disease progression in individuals. Participant 1, for example, shows a sharp increase in $\lambda^{*}$ halfway between the stem and the present (Fig. 8G and H), indicative of an increase in positive selection in the virus. This is followed by a drop in *ψ* at the next time-slice, showing that the increase in $\lambda^{*}$ is succeeded by a slowing down in diversification rate, a pattern that is repeated leading to the present (Fig. 8H). The immediate decrease in *η* between the first two time-slices shows a sharp rise in mutational fitness following the initial sampling. This rise is expected, but its extent (i.e. difference in *η*) appears to be idiosyncratic to each individual and therefore may be diagnostic of the initial diversification of the infection. We furthermore tested for any differences in phylogenetic diversification among the HIV-1 sequences which were predicted to use the CXCR4 coreceptor for viral entry (Shankarappa et al. 1999). We found that X4 variants typically had larger $\lambda^{*}$ and lower *η* values at each timepoint (Supplementary Fig. S3), but that this was not significant, possibly due to restrictively small effect sizes (Cohen’s D=0.18±0.07).

## 3. Discussion

We presented the spectral density profile of the MGL as a new tool for clustering and characterizing viral phylogenies. It enables the user to survey population-wide patterns of within-host viral diversification and test the effects of epidemiological factors on those patterns, thereby providing an additional option to help overcome some of the difficulties of using parametric approaches for inferring viral evolution (Suzuki and Nei 2004; Duchêne et al. 2015). We used three examples to illustrate some of the features of the MGL.

The spectral density profile of the MGL can characterize diversification dynamics within hosts and compare them between hosts under a multidimensional framework with a rigorous theoretical basis. It can be computed rapidly for reasonably sized phylogenies (9 seconds for a tree with 1,000 tips), which is valuable for initial probes into the structure of big data that can then be integrated with existing metrics (e.g. genetic divergence, dN/dS, and codon frequencies). One key contribution of this approach to the phylodynamics toolbox is its ability to identify clusters of individuals based on their viral diversification dynamics. Once identified, these clusters can be characterized by spectral density summary statistics, which represent distinct aspects of phylogenetic diversification: $\lambda^{*}$ is an estimate of the maximum evolutionary change in a phylogeny, which is distinct from estimates of total diversity; *ψ* is the proportion of shorter *versus* longer branches, which is a measure of the extent of rate heterogeneity through time and across lineages; *η* operates as a complement to $\lambda^{*}$, as it accounts for the degree of mutational fitness in the evolutionary change in a phylogeny, where A↔G and C↔T transitions are more fit than A↔C,A↔T,C↔G, and G↔T transversions; and the eigengap, a measure of disconnectedness in a network, reflects the elapsed time between sampling in longitudinal data. Because no virus evolves under a single selection pressure, different selection pressures are expected to have different effects. Using hypothesis-based comparisons of spectral density profile summary statistics on data from the RV144 trial (Rolland et al. 2012), we identified no effect of sex, treatment, days since infection, or RNA copies/ml, whereas, using unbiased clustering on spectral density profiles, we found that founder heterogeneity distinguished diversification patterns among participants. Hence, the MGL approach is a rapid approach to evaluate phylogenies in light of a variety of attributes, both discrete (e.g. co-infection, risk group, geography) and continuous (e.g. neutralization breadth, CD4+ T-cell count).

Contemporary sequences from the transmitter are typically not available when analyzing sequences from newly infected individuals. Yet, identifying whether the new infection was established with a single variant or multiple variants from the transmitter remains important. Traditionally, in the absence of sequences from the transmitter, founder heterogeneity has been determined using a combination of qualitative measures (visual inspections of highlighter plots and tree topologies), quantitative measures of diversity (intra-host pairwise; number of shared *versus* private mutations), and by testing the goodness-of-fit to a Poisson-model (Keele et al. 2008; Abrahams et al. 2009; Haaland et al. 2009; Herbeck et al. 2011; Rolland et al. 2011, 2012; Janes et al. 2015; Tully et al. 2016). In the Keele et al. (2008) dataset, which we analyzed here, the authors define founder heterogeneity by a best-fitting Poisson distribution of Hamming distances determined by a model parameterized by assumptions on phylogenetic topology, HIV-1 generation time, reproductive ratio, reverse transcriptase point mutation rate, and infection rate. Here we define a heuristic test for classifying founder heterogeneity based on the principal eigenvalue, $\lambda^{*}$, of the MGL. We show that this can efficiently distinguish homogeneous/single from heterogeneous/multiple founders in the Keele et al. (2008) dataset. We propose a threshold for heterogeneous founders at the median $+n\sigma^{2}$ of $\lambda^{*}$, for *n* = 1/2. However, given the flexibility of the test to define the threshold within a confidence interval, the user is free to consider a different value for *n* or implement one of the other techniques (the jump or partition) for establishing a threshold. By giving a confidence interval (defined by $\pm n\sigma^{2}$) to the threshold between homogeneous and heterogeneous founders, we can consider founder heterogeneity along a continuum, rather than as a binary trait.

How a viral infection evolves in an individual over time may be demonstrative of how predictably a disease typically progresses, but may also reveal differences in disease progression between individuals. Using sequences from HIV-1-infected males sampled from the time of seroconversion to the development of advanced disease, we charted the progressive change in phylogenetic diversification of the virus in each participant. We showed that it is possible to identify both general patterns of disease progression over time using the spectral density profile, as well as deviations from those general patterns particular to each participant. For example, in all participants, $\lambda^{*}$ increased at each time-step, but the rate at which it increased was unique to each participant. Likewise, *η* followed a negative logarithmic trend towards the present for all participants, although the slope parameter defining when the value reached its minimum (i.e. the elbow) varied between participants. This elbow generally corresponded to the first major *ψ* peak in participants. As *η* is an inverse measure of mutational fitness and *ψ* corresponds to increases in rate heterogeneity between samples, these patterns—the first *ψ* peak and elbow of *η*—suggest the time at which the virus in each participant underwent a shift in diversification. This is consistent with the original analysis of these data (Shankarappa et al. 1999), which showed that peak within-timepoint diversity coincides with the first *ψ* peak and elbow of *η*. Thus, the spectral density profiles of time-sliced within-host phylogenies can help measure complex patterns of disease progression in individuals and across populations (and diseases) that are typically not captured (Hill et al. 2018).

## 4. Conclusions

We have described how the spectral density profile of the MGL can be applied to viral phylogenies. We show that important features of molecular evolution and phylogenetic diversification are retained in the profiles of viral trees and that this is an efficient approach for analyzing within-host evolution and for classifying founder multiplicity of infections. Of course, there are limitations to our approach and critical aspects of viral evolution and diversification that the spectral density profile does not capture. First, our approach is sensitive to phylogenetic reconstruction, as evidenced by the differences in spectral density profile summary statistics in phylogenies constructed with a Bayesian *versus* a maximum-likelihood framework. Therefore, it is important that tree construction is consistent across samples and that comparison between individuals and cohorts be explicit about how trees are constructed. Second, while $\lambda^{*}$ captures evidence of a signature of positive selection, it cannot identify which regions of the gene are specifically being targeted for selection (although, comparisons between phylogenies constructed for different genes could reveal differences in selection pressures; Lewitus and Morlon 2016a). Further to this point, it is important that users follow general guidelines of good practice when using the spectral density profile: the MGL of a phylogeny is necessarily sensitive to effects of recombination and time since infection; we advise that users test for recombination (Kosakovsky Pond et al. 2006). Finally, clustering spectral density profiles of viral phylogenies from individuals does not account for the transmission chain of the virus, which, if molecular aspects of the virus are heritable, may have an impact on diversification dynamics within each individual (Felsenstein 1973; Mitov and Stadler 2018).

Although this article has focused on HIV-1 datasets and applications, we present it as a general approach that is valid for analyzing other viruses. We think that the spectral density profile of the MGL is an important addition to the increasingly accessible set of analytic and programmatic tools for investigating viral diversification dynamics.

## 5. Materials and methods

### 5.1 Simulating molecular evolution and longitudinal trees

We simulated samples of 10 sequences under a GY94 codon substitution model for 600 nucleotides. We simulated three scenarios (200 samples each) with one changing parameter: a non-synonymous to synonymous substitution rate ratio (dN/dS) set to 0.1, 0.7, or 1.3; a normal, discrete, or invariantgeneralised time-reversible (GTR) nucleotide substitution model (*γ* distribution); and a Hasegawa–Kishino–Yano (HKY) nucleotide substitution model with a transition-to-transversion rate ratio (ti/tv) set to 50/1, 25/25, or 1/50. We also simulated permutations of all three parameters combined (200 samples each): dN/dS = 0.1, 0.7; *γ* distribution = discrete, invariant; and ti/tv = 1/50, 50/1. Simulations were run with a Monte Carlo sequence simulator adapted from an exact stochastic model (Gillespie 1977; Sipos et al. 2011). Each sample of sequences was aligned using an iterative refinement method in MAFFT v.7 (Katoh and Standley 2013) that incorporates local pairwise alignment information; and phylogenetic trees were constructed from the aligned sequences using IQ-TREE (Nguyen et al. 2015). We computed the spectral density profiles for the trees from the weighted modified graph Laplacian of their distance matrices, the so-called MGL (Lewitus and Morlon, 2016a; Morlon et al. 2016). Summary statistics of the spectral density profiles were measured as the principal eigenvalue ($\lambda^{*}$), skewness of the profile (*ψ*), and the peak height of the profile (*η*). For the multiple parameter trees, we clustered profiles based on Jensen-Shannon distances (Endres and Schindelin 2003) using hierarchical clustering with bootstrap probabilities calculated at each node and an optimal number of supported clusters determined by partitioning around medoids (Reynolds et al. 2006).

We simulated pure-birth trees with two sampling time-points and a constant speciation rate (0.1). We simulated 200 trees each with a sampling time of 1–29 days at 7-day intervals and at 5–40 weeks at 5-week intervals (for a total of 2,800 trees). The sampling fraction for each simulated tree was 0.1. We computed the spectral density profile for each tree. We ranked the *λ* of the MGL for each tree from largest to smallest and determined the position of the so-called eigengap, which is defined as the largest difference between two ranked eigenvalues, *λ_i_* and $\lambda_{i+1}$ (Von Luxburg 2007). Because each *λ* is a measure of the connectivity of a graph (i.e. tree), disproportionately large *λ* represent a near-disconnection between cells in the graph (i.e. branches in the tree). Therefore, the eigengap is an indicator of the number of disconnected groups of branches in the tree (Shen and Cheng 2010), where an eigengap between *λ_i_* and $\lambda_{i+1}$ indicates *i* near-disconnected groups of branches (i.e. clusters). We calculated the value of the eigengap for each tree as the distance between *λ_i_* and $\lambda_{i+1}$. We additionally estimated the maximum pairwise genetic dissimilarity for each tree.

We simulated pure-birth trees with two sampling time-points. We simulated 200 trees with a constant speciation rate (0.1) at the first time-point and a speciation rate increase of 0.01, 0.2, 0.4, 0.6, 0.8 at the second time-point (for a total of 1,000 trees). The time between sampling was set to 10 weeks. We computed the spectral density profile summary statistics and the maximum pairwise genetic similarity for each tree.

Trees were simulated using the *R* package *TESS* (Höhna, May, and Moore 2015). Sequence data were simulated on trees using the simSeq function in the *R* package *phangorn* (Schliep 2011; Benidt and Nettleton 2015; Schliep et al. 2017) for sequence lengths of 600, uniform base frequencies, and a GTR rate matrix.

### 5.2 Hypothesis-testing and unbiased clustering with participants from the RV144 cohort

We downloaded 936 HIV-1 *env* sequences sampled at diagnosis from individuals in the RV144 trial with CRF01_AE (Rolland et al. 2012). We aligned sequences with an iterative refinement algorithm in MAFFT v.7 (Katoh and Standley 2013) that incorporates local pairwise alignment information for individuals with at least 10 samples, resulting in multiple alignments for 110 individuals. We constructed phylogenies for each individual with IQ-TREE (Nguyen et al. 2015), using ModelFinder to infer the model with the smallest Bayesian information criterion score (Kalyaanamoorthy et al. 2017), and assessed node support by 1,000 ultrafast bootstrap replicates (Minh, Nguyen, and von Haeseler 2013). We computed spectral density profiles for phylogenies as above.

We subset the 110 individuals by sex, treatment, and founder heterogeneity according to accompanying metadata and compared subsets using one-sample t-tests. We estimated the effect of each summary statistic on different infection factors (days since last negative test, days since Day 0, and RNA copies/ml) by fitting ordinary least squares (OLS) regressions. We additionally clustered the phylogenies using Jensen-Shannon distances of their spectral density profiles by partitioning around medoids and determined the optimal number of clusters based on Duda-Hart tests (Duda, Hart, and Stork 1973).

### 5.3 A heuristic test for founder heterogeneity with *env* sequences from participants acutely infected with HIV-1

We downloaded aligned per-individual fasta files from the Los Alamos National Laboratory HIV sequence database (www.hiv.lanl.gov) for seventy-two individuals with acute HIV-1 infections, including fifty-three with low-diversity *env* sequences that conformed to a model of random evolution in early infection and nineteen with high-diversity *env* sequences that did not conform to a model of random evolution in acute infection due to infection by more than one divergent strain (Keele et al. 2008). We did not include participants that contained hypermutated sequences or more than one related founder strain. We removed the consensus sequence from each file and then realigned them before constructing trees with IQ-TREE as above. In total, we constructed trees and spectral density profiles for *env* sequences sampled from seventy-two HIV-1-infected individuals and compared summary statistics for individuals with an acute infection from one (homogeneous) or more than one (heterogeneous) virus. We furthermore constructed trees from *env* sequences for the same samples using BEAST v2.5.2 (Bouckaert et al. 2014) with a HKY substitution model, an uncorrelated log-normal relaxed clock, the substitution rate prior set with a mean per-day rate of 2.24e−5 and a uniform prior for effective population size between 1−1e10 (Lemey, Rambaut, and Pybus 2006). For each tree, we ran 1e7 generations and a burn-in rate of 10%. Only trees that converged within 1e7 generations were analyzed. Analyses on BEAST trees were conducted on majority rule consensus trees estimated with TreeAnnotator (Bouckaert et al. 2014). Spectral density profiles were computed for BEAST trees as above.

We designed three tests for distinguishing participants with homogeneous and heterogeneous founder pools based on ln-transformed $\lambda^{*}$. (1) We defined a threshold based on the median value $+n\sigma^{2}$ of $\lambda^{*}$. Here, the value of *n* determines the confidence we can assign to participants above the median as having heterogeneous founder pools. (2) We define the threshold at the largest distance (or ‘jump‘) between ranked $\lambda^{*}$. (3) We partition spectral density profiles around medoids, which minimizes a sum of dissimilarities, assuming two clusters (Reynolds et al. 2006; Schubert and Rousseeuw 2019; Maechler et al. 2019).

### 5.4 Time-slicing phylogenies from participants in the multicenter AIDS Cohort Study

We downloaded the 1,300 aligned sequences sampled from nine participants as part of the Multicenter AIDS Cohort Study (Kaslow et al. 1987; Shankarappa et al. 1999) from the Los Alamos National Laboratory HIV sequence database (www.hiv.lanl.gov). We separated the sequences by participant, realigned them using an iterative refinement algorithm in MAFFT v.7 (Katoh and Standley 2013) that incorporates local pairwise alignment information, and constructed phylogenies for the new alignments using IQ-TREE (Nguyen et al. 2015). We then sliced each phylogeny at nine time-points beginning at 20% of the distance between the root and the present, where 100% is the entire phylogeny. We decided on nine time-points, because it was the largest number of slices wherein each slice had a different number of lineages for all phylogenies. We then computed spectral density profile summary statistics for each slice of each phylogeny as outlined above.

## Supplementary Material

vez044_Supplementary_Data
