# Supplementary material for: A non-parametric analytic framework for within-host viral phylogenies and a test for HIV-1 founder multiplicity
Source: Virus Evol. 2019 Nov 4;5(2):vez044. doi: 10.1093/ve/vez044 (PMC6826062; doi:10.1093/ve/vez044)
Supplement: vez044_Supplementary_Data [file vez044_supplementary_data.zip › SupplementalTable1_Caption.docx]

Supplemental Table 1: Ln-transformed principal eigenvalues and inferred founder multiplicity for trees in the Keele et al., 2008 dataset using the principal eigenvalue test of founder multiplicity.
