# Supplementary material for: A non-parametric analytic framework for within-host viral phylogenies and a test for HIV-1 founder multiplicity
Source: Virus Evol. 2019 Nov 4;5(2):vez044. doi: 10.1093/ve/vez044 (PMC6826062; doi:10.1093/ve/vez044)
Supplement: vez044_Supplementary_Data [file vez044_supplementary_data.zip › FigureS1.pdf]

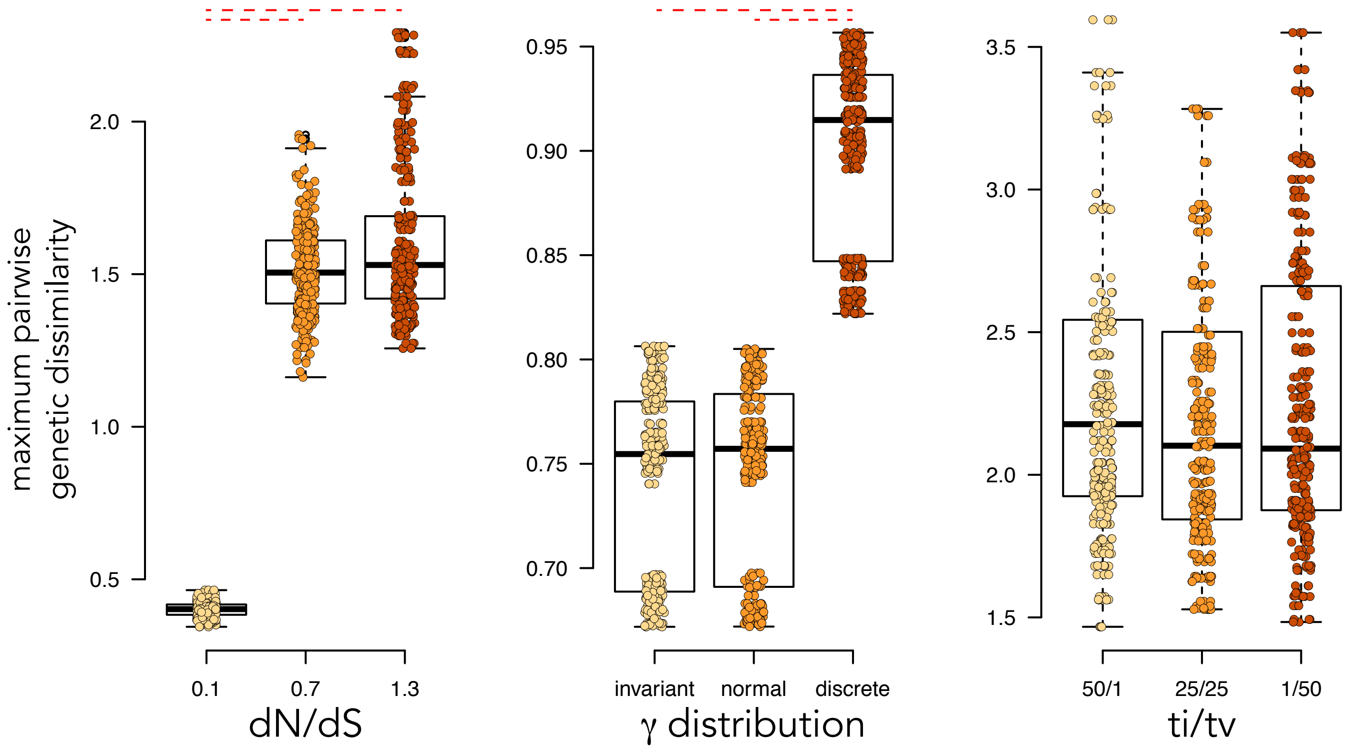
maximum pairwise
genetic dissimilarity
2.0
1.5
1.0
0.5
0.1
0.7
1.3
dN/dS
0.95
0.90
0.85
0.80
0.75
0.70
invariant
normal
discrete
γ distribution
3.5
3.0
2.5
2.0
1.5
50/1
25/25
1/50
ti/tv
