# Supplementary figures and images for: A non-parametric analytic framework for within-host viral phylogenies and a test for HIV-1 founder multiplicity
Source: Virus Evol. 2019 Nov 4;5(2):vez044. doi: 10.1093/ve/vez044 (PMC6826062; doi:10.1093/ve/vez044)

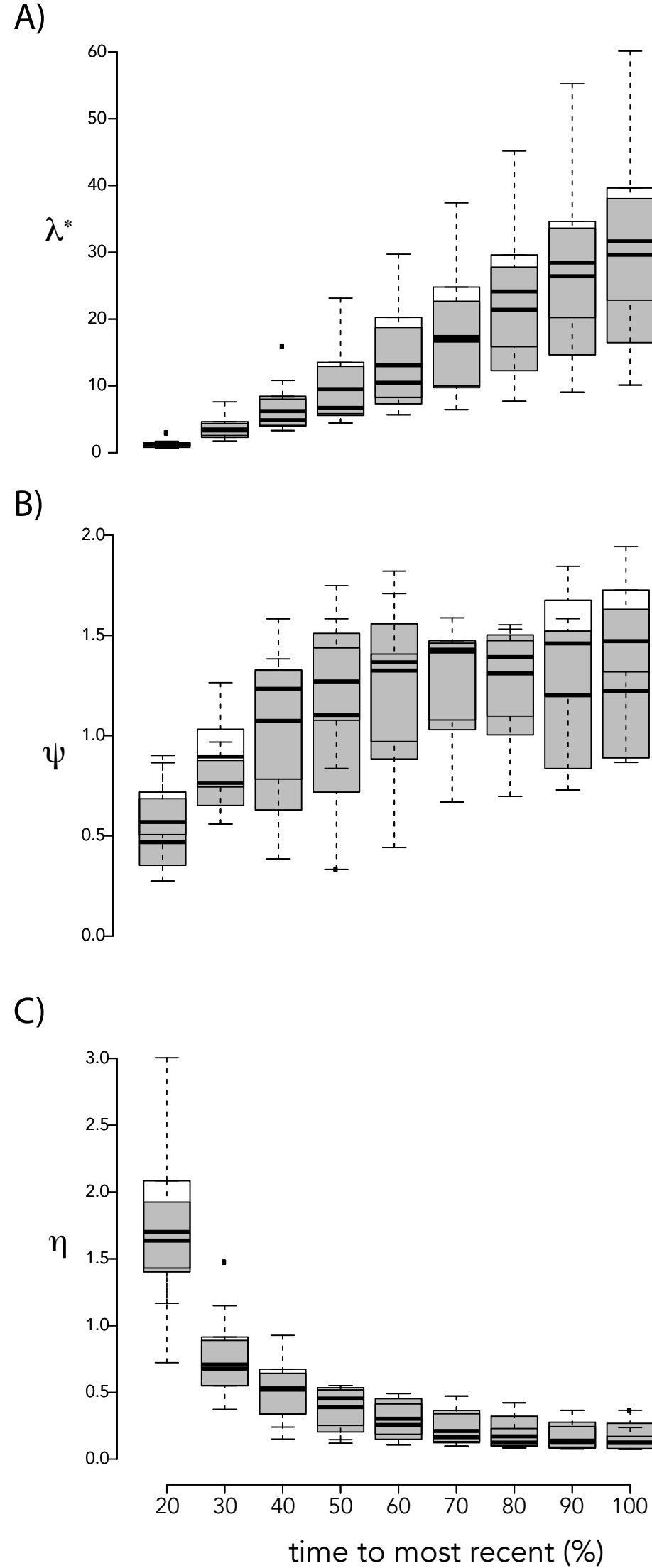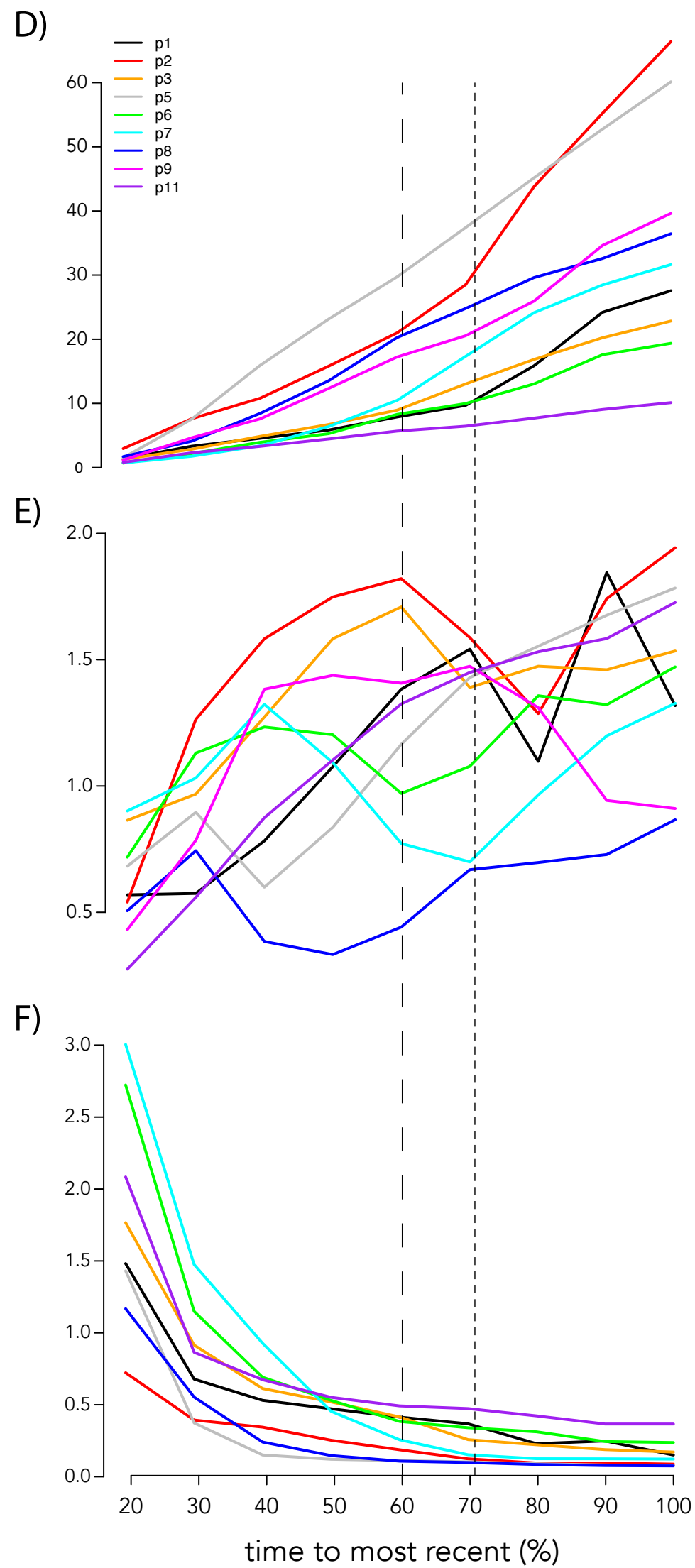

Supplement: vez044_Supplementary_Data [file vez044_supplementary_data.zip › FigureS3.pdf]

A)

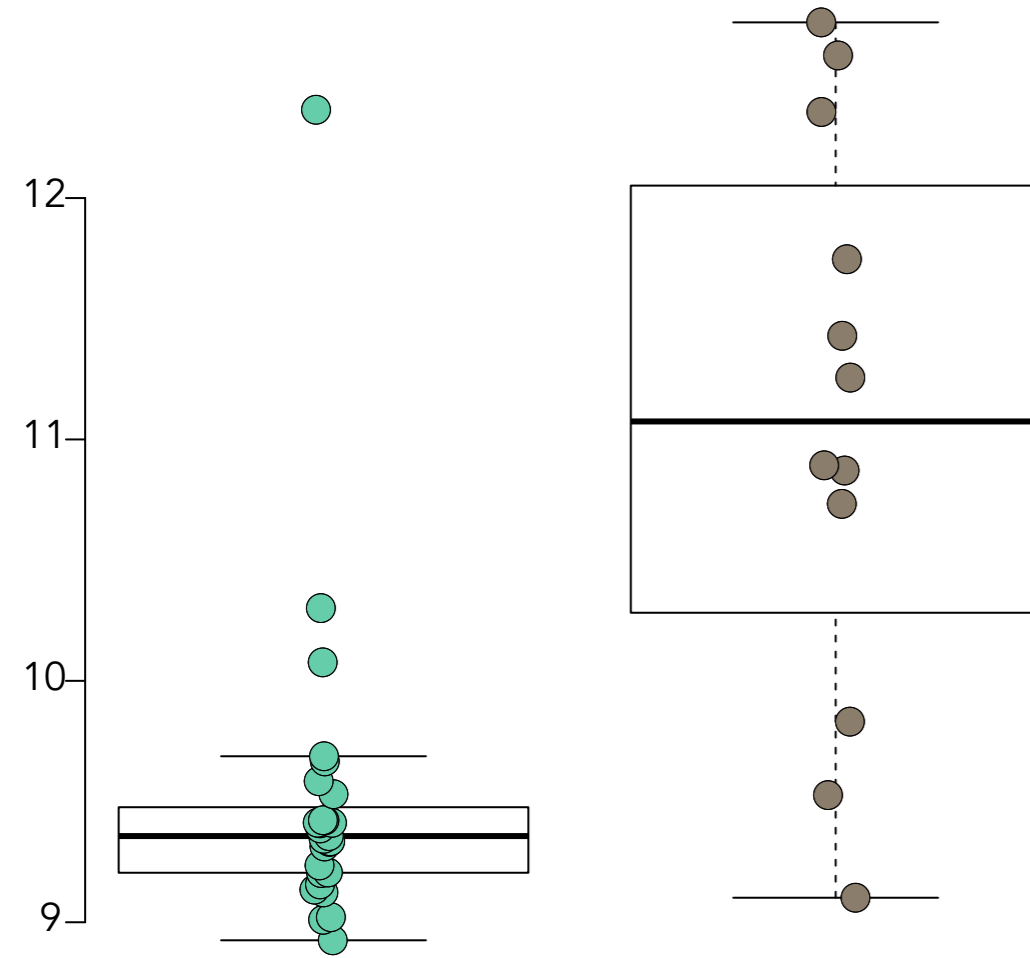

B)

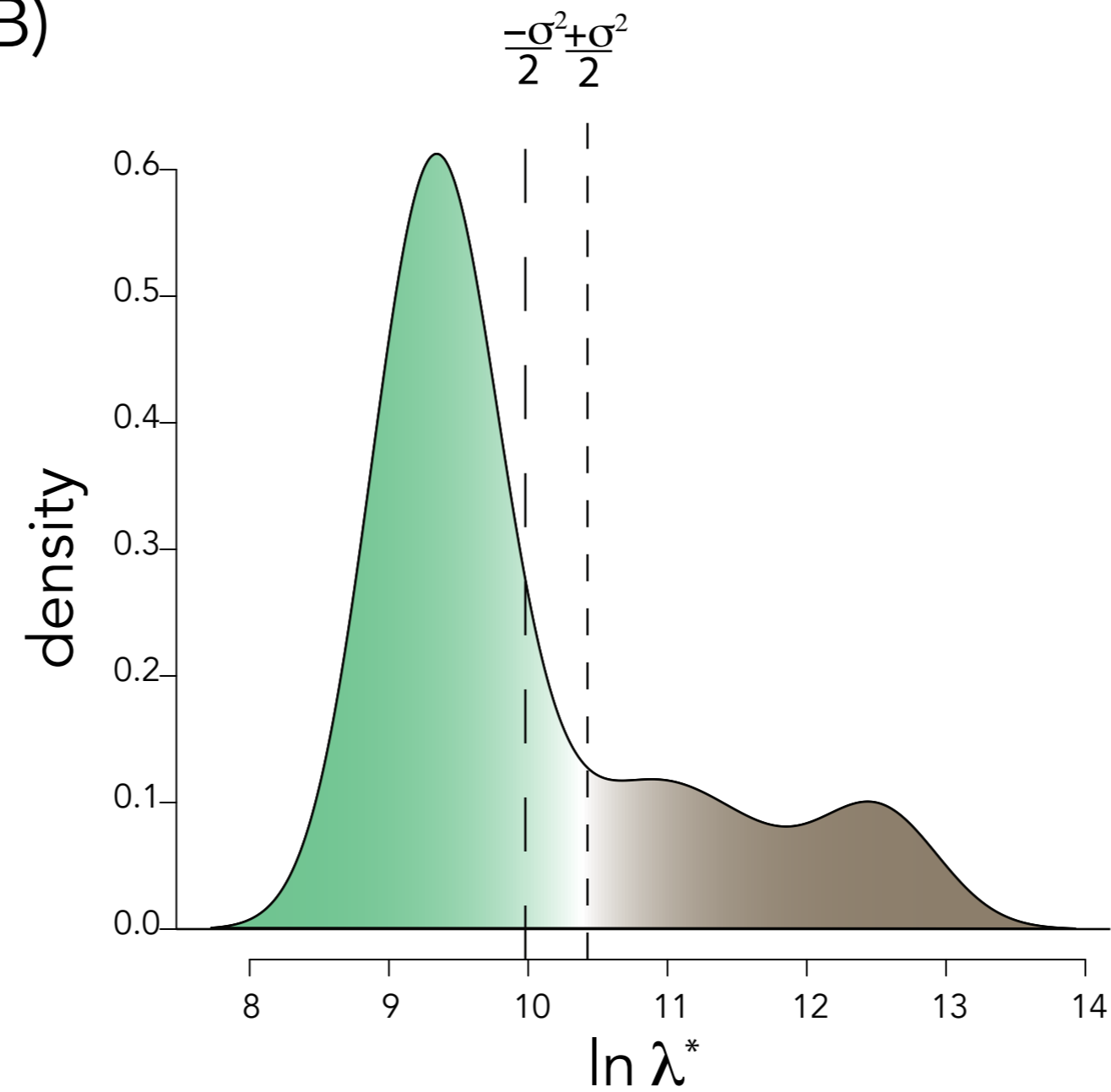

C)

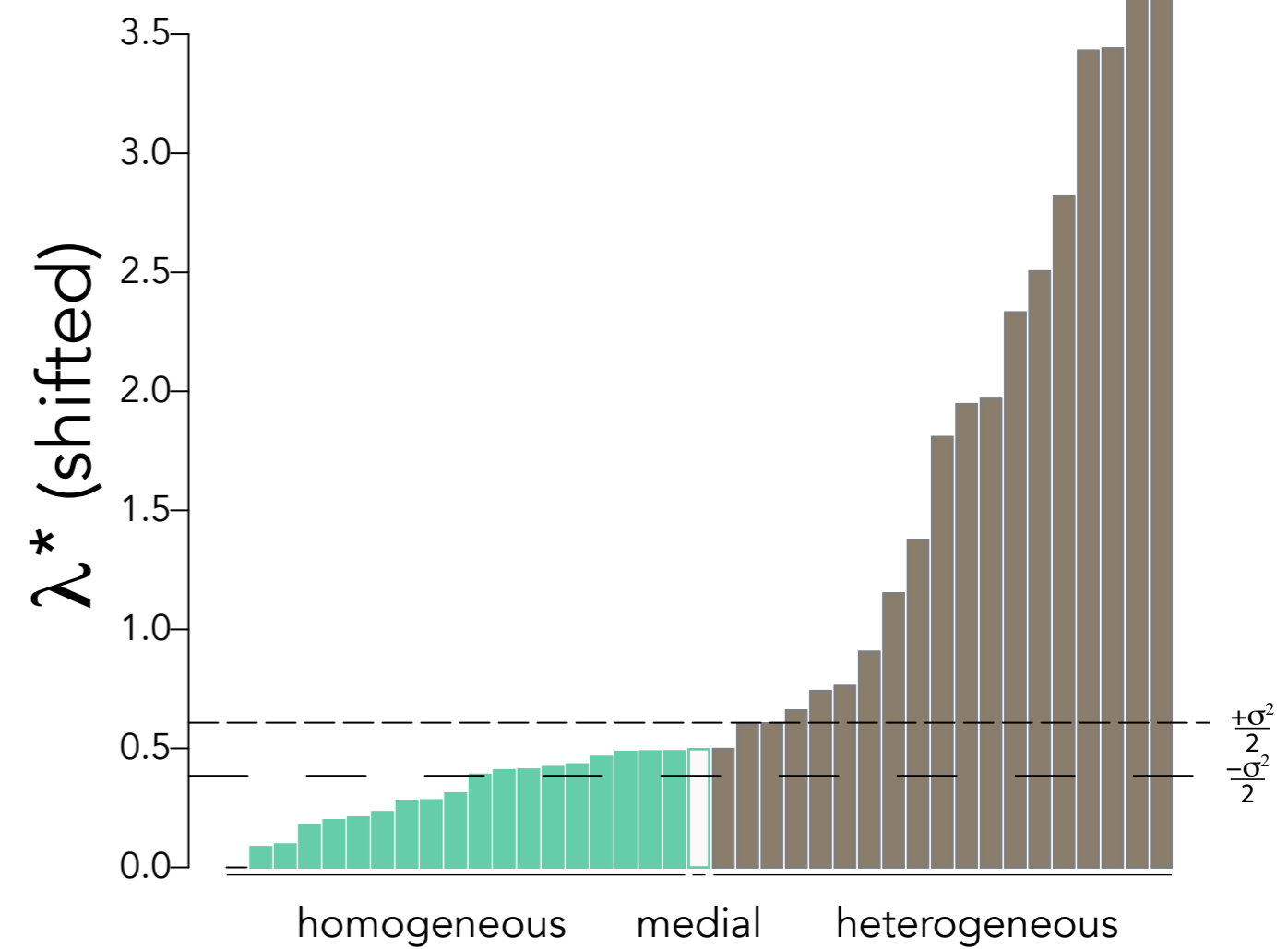

Supplement: vez044_Supplementary_Data [file vez044_supplementary_data.zip › FigureS2.pdf]
